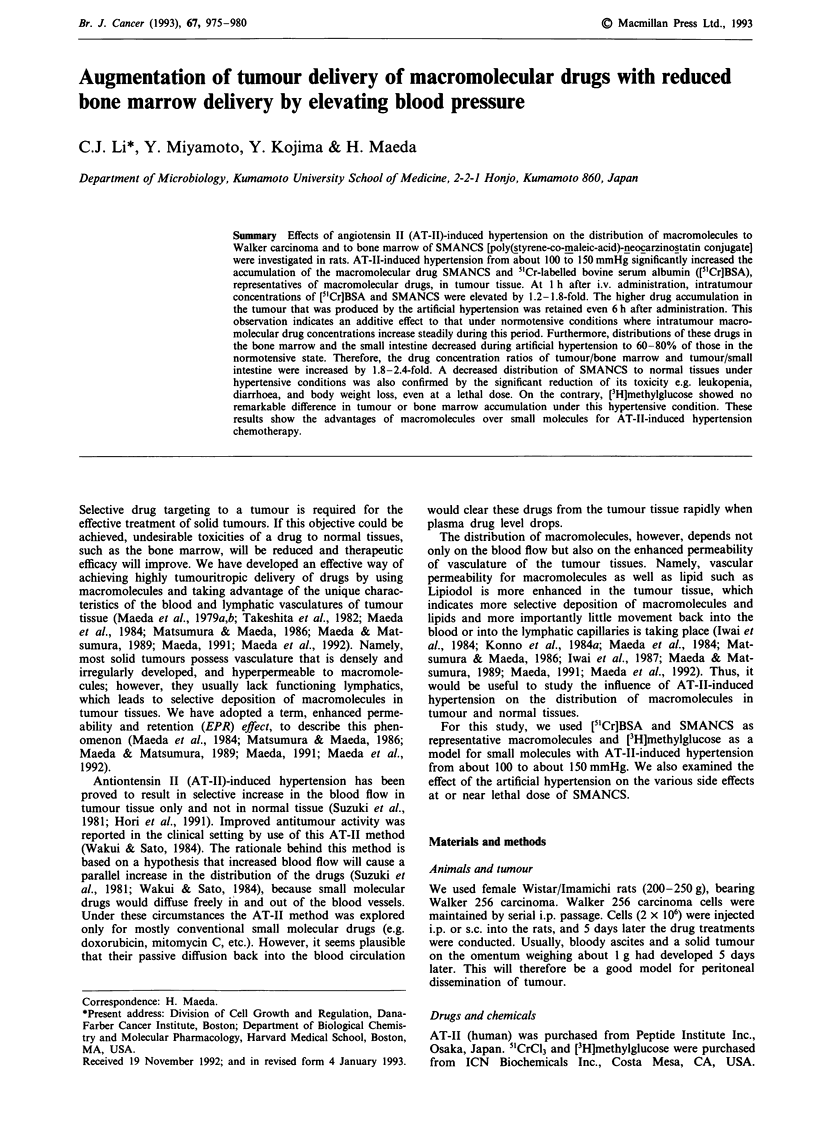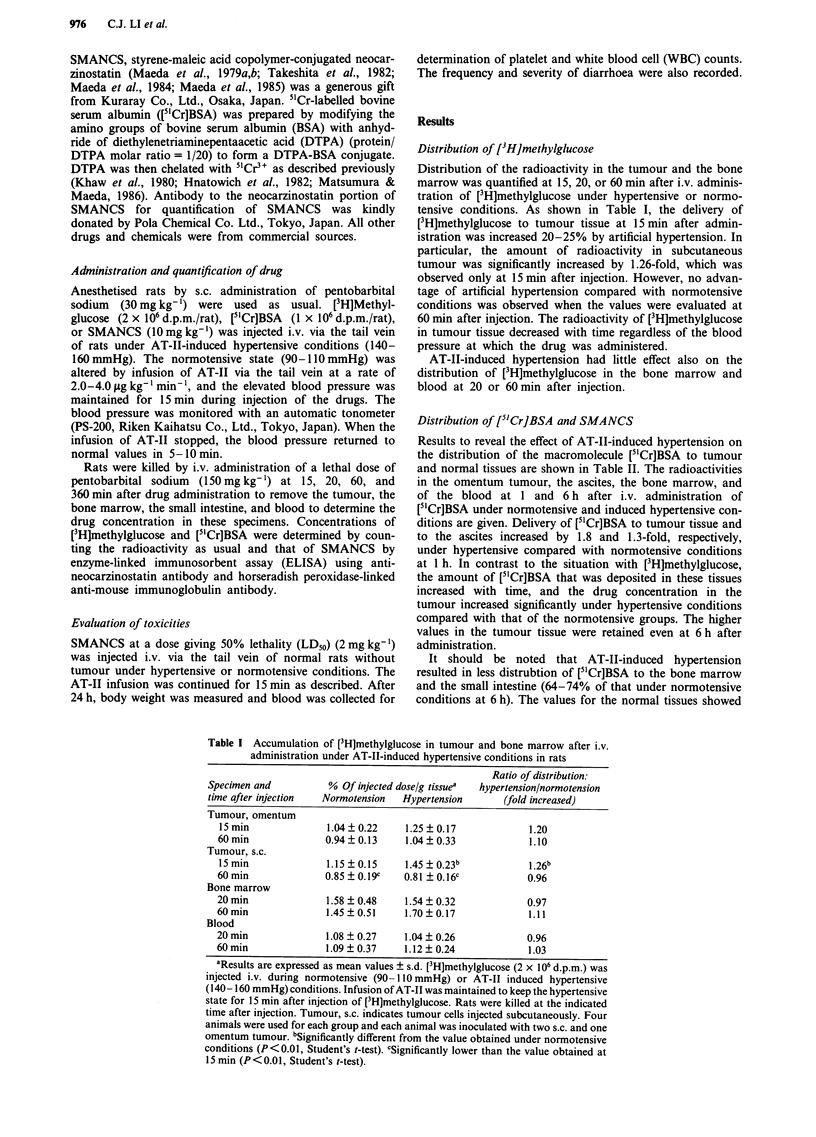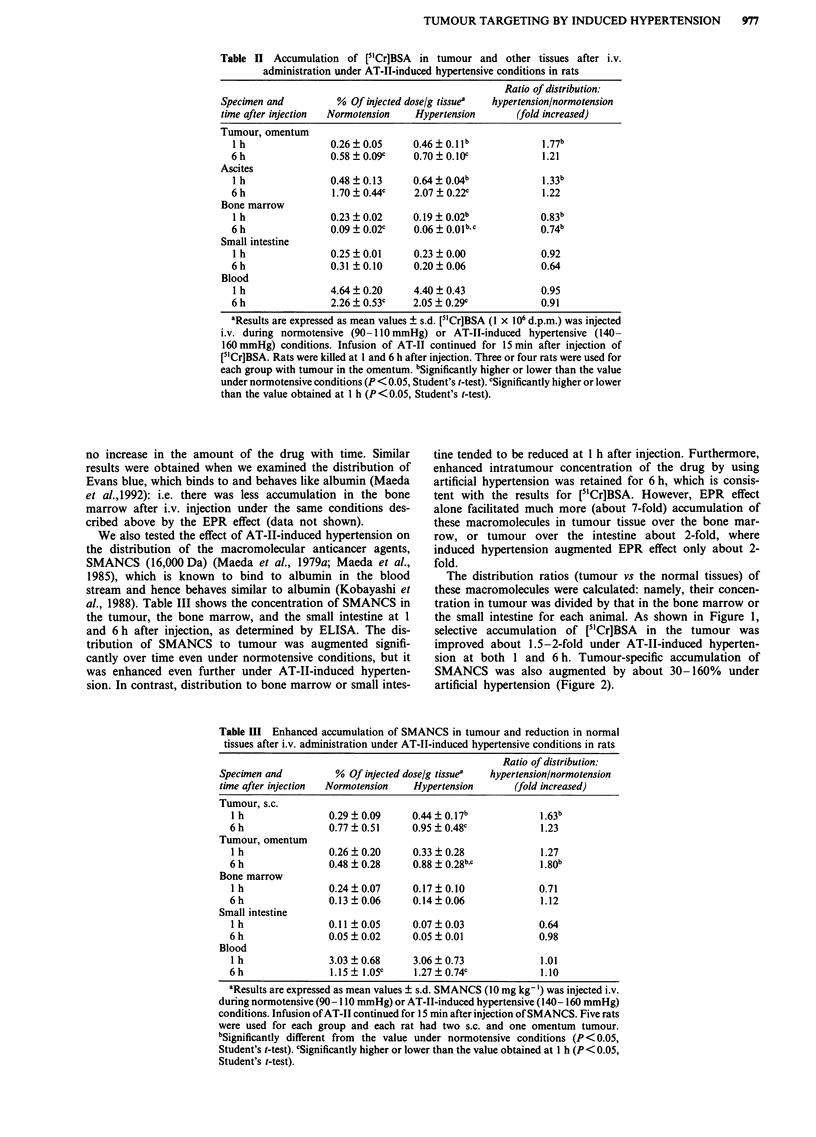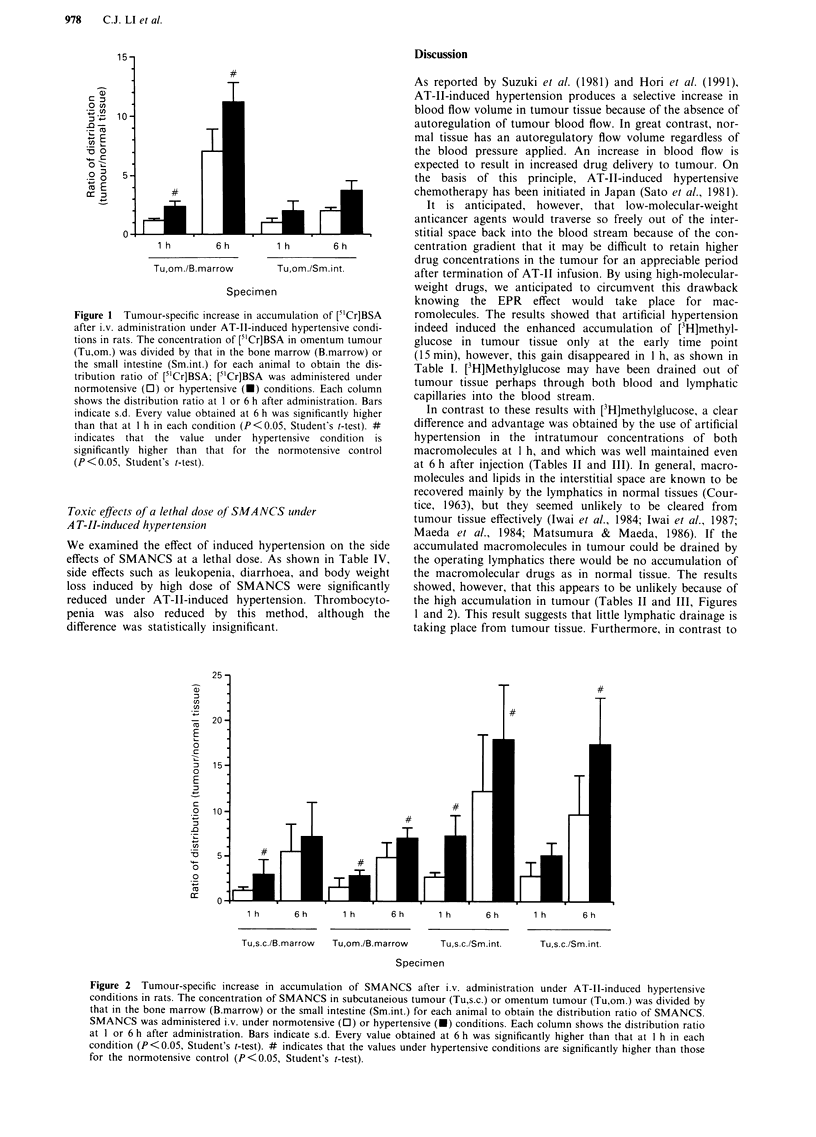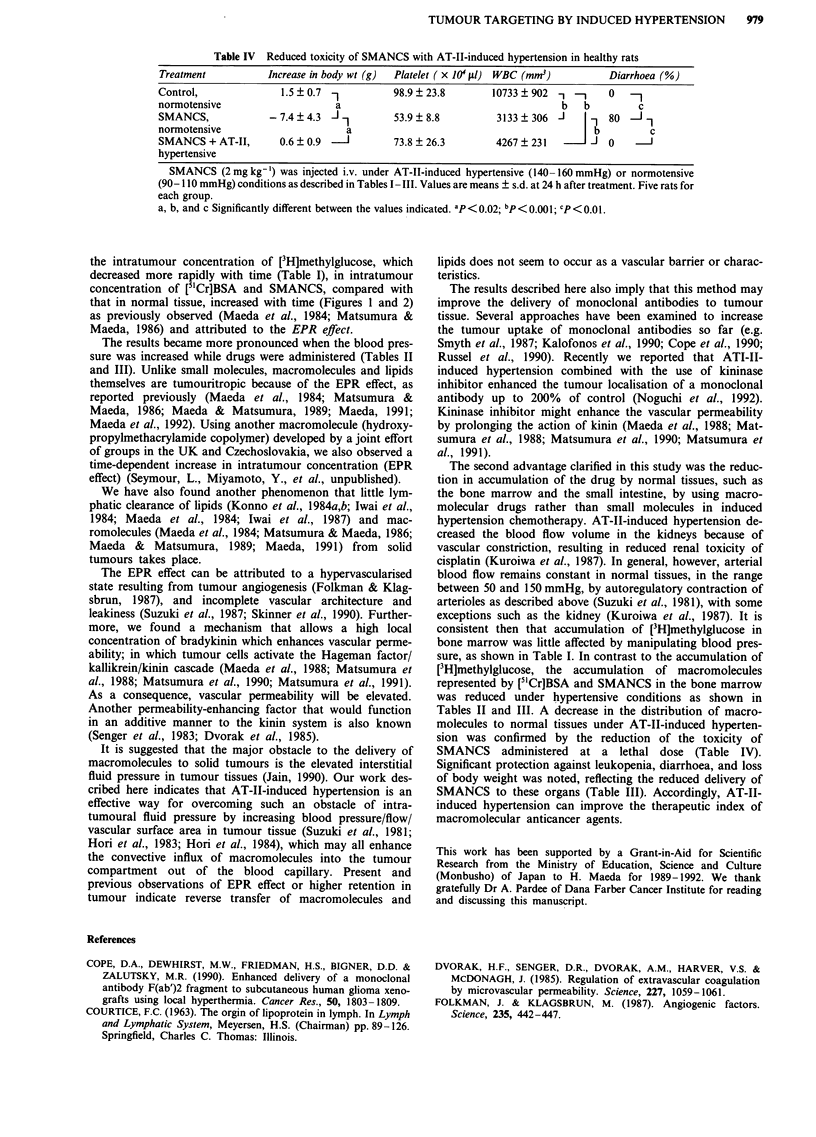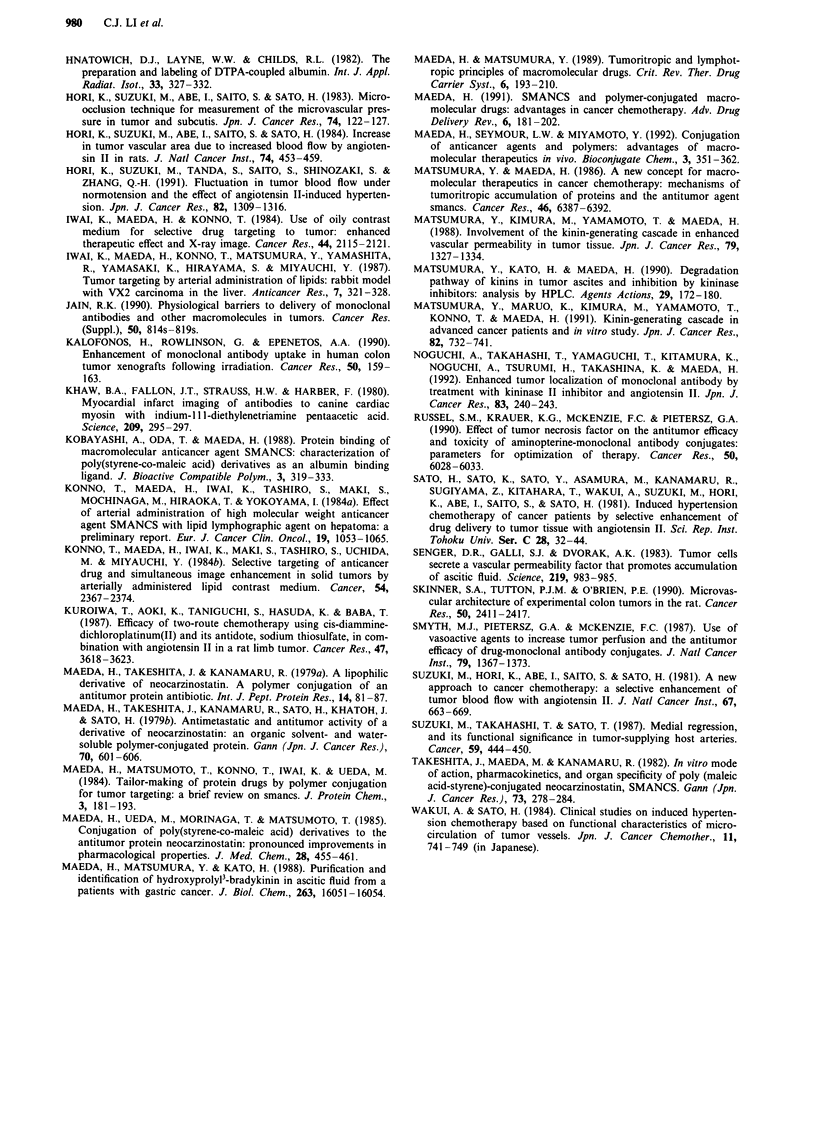# Augmentation of tumour delivery of macromolecular drugs with reduced bone marrow delivery by elevating blood pressure.

**DOI:** 10.1038/bjc.1993.179

**Published:** 1993-05

**Authors:** C. J. Li, Y. Miyamoto, Y. Kojima, H. Maeda

**Affiliations:** Department of Microbiology, Kumamoto University School of Medicine, Japan.

## Abstract

Effects of angiotensin II (AT-II)-induced hypertension on the distribution of macromolecules to Walker carcinoma and to bone marrow of SMANCS [poly(styrene-co-maleic-acid)-neocarzinostatin conjugate] were investigated in rats. AT-II-induced hypertension from about 100 to 150 mmHg significantly increased the accumulation of the macromolecular drug SMANCS and 51Cr-labelled bovine serum albumin ([51Cr]BSA), representatives of macromolecular drugs, in tumour tissue. At 1 h after i.v. administration, intratumour concentrations of [51Cr]BSA and SMANCS were elevated by 1.2-1.8-fold. The higher drug accumulation in the tumour that was produced by the artificial hypertension was retained even 6 h after administration. This observation indicates an additive effect to that under normotensive conditions where intratumour macromolecular drug concentrations increase steadily during this period. Furthermore, distributions of these drugs in the bone marrow and the small intestine decreased during artificial hypertension to 60-80% of those in the normotensive state. Therefore, the drug concentration ratios of tumour/bone marrow and tumour/small intestine were increased by 1.8-2.4-fold. A decreased distribution of SMANCS to normal tissues under hypertensive conditions was also confirmed by the significant reduction of its toxicity e.g. leukopenia, diarrhoea, and body weight loss, even at a lethal dose. On the contrary, [3H]methylglucose showed no remarkable difference in tumour or bone marrow accumulation under this hypertensive condition. These results show the advantages of macromolecules over small molecules for AT-II-induced hypertension chemotherapy.


Br. J. Cancer (1993), 67, 975-980 © Macmillan Press Ltd., 1993

### Augmentation of tumour delivery of macromolecular drugs with reduced bone marrow delivery by elevating blood pressure

C.J. Li*, Y. Miyamoto, Y. Kojima & H. Maeda

*Department of Microbiology, Kumamoto University School of Medicine, 2-2-1 Honjo, Kumamoto 860, Japan*

**Summary** Effects of angiotensin II (AT-II)-induced hypertension on the distribution of macromolecules to Walker carcinoma and to bone marrow of SMANCS [poly(styrene-co-maleic-acid)-neocarzinostatin conjugate] were investigated in rats. AT-II-induced hypertension from about 100 to 150 mmHg significantly increased the accumulation of the macromolecular drug SMANCS and $^{51}$Cr-labelled bovine serum albumin ([$^{51}$Cr]BSA), representatives of macromolecular drugs, in tumour tissue. At 1 h after i.v. administration, intratumour concentrations of [$^{51}$Cr]BSA and SMANCS were elevated by 1.2-1.8-fold. The higher drug accumulation in the tumour that was produced by the artificial hypertension was retained even 6 h after administration. This observation indicates an additive effect to that under normotensive conditions where intratumour macromolecular drug concentrations increase steadily during this period. Furthermore, distributions of these drugs in the bone marrow and the small intestine decreased during artificial hypertension to 60-80% of those in the normotensive state. Therefore, the drug concentration ratios of tumour/bone marrow and tumour/small intestine were increased by 1.8-2.4-fold. A decreased distribution of SMANCS to normal tissues under hypertensive conditions was also confirmed by the significant reduction of its toxicity e.g. leukopenia, diarrhoea, and body weight loss, even at a lethal dose. On the contrary, [$^{3}$H]methylglucose showed no remarkable difference in tumour or bone marrow accumulation under this hypertensive condition. These results show the advantages of macromolecules over small molecules for AT-II-induced hypertension chemotherapy.

Selective drug targeting to a tumour is required for the effective treatment of solid tumours. If this objective could be achieved, undesirable toxicities of a drug to normal tissues, such as the bone marrow, will be reduced and therapeutic efficacy will improve. We have developed an effective way of achieving highly tumouritropic delivery of drugs by using macromolecules and taking advantage of the unique characteristics of the blood and lymphatic vasculatures of tumour tissue (Maeda *et al.*, 1979*a,b*; Takeshita *et al.*, 1982; Maeda *et al.*, 1984; Matsumura & Maeda, 1986; Maeda & Matsumura, 1989; Maeda, 1991; Maeda *et al.*, 1992). Namely, most solid tumours possess vasculature that is densely and irregularly developed, and hyperpermeable to macromolecules; however, they usually lack functioning lymphatics, which leads to selective deposition of macromolecules in tumour tissues. We have adopted a term, enhanced permeability and retention (*EPR*) *effect*, to describe this phenomenon (Maeda *et al.*, 1984; Matsumura & Maeda, 1986; Maeda & Matsumura, 1989; Maeda, 1991; Maeda *et al.*, 1992).

Antiontensin II (AT-II)-induced hypertension has been proved to result in selective increase in the blood flow in tumour tissue only and not in normal tissue (Suzuki *et al.*, 1981; Hori *et al.*, 1991). Improved antitumour activity was reported in the clinical setting by use of this AT-II method (Wakui & Sato, 1984). The rationale behind this method is based on a hypothesis that increased blood flow will cause a parallel increase in the distribution of the drugs (Suzuki *et al.*, 1981; Wakui & Sato, 1984), because small molecular drugs would diffuse freely in and out of the blood vessels. Under these circumstances the AT-II method was explored only for mostly conventional small molecular drugs (e.g. doxorubicin, mitomycin C, etc.). However, it seems plausible that their passive diffusion back into the blood circulation would clear these drugs from the tumour tissue rapidly when plasma drug level drops.

The distribution of macromolecules, however, depends not only on the blood flow but also on the enhanced permeability of vasculature of the tumour tissues. Namely, vascular permeability for macromolecules as well as lipid such as Lipiodol is more enhanced in the tumour tissue, which indicates more selective deposition of macromolecules and lipids and more importantly little movement back into the blood or into the lymphatic capillaries is taking place (Iwai *et al.*, 1984; Konno *et al.*, 1984*a*; Maeda *et al.*, 1984; Matsumura & Maeda, 1986; Iwai *et al.*, 1987; Maeda & Matsumura, 1989; Maeda, 1991; Maeda *et al.*, 1992). Thus, it would be useful to study the influence of AT-II-induced hypertension on the distribution of macromolecules in tumour and normal tissues.

For this study, we used [$^{51}$Cr]BSA and SMANCS as representative macromolecules and [$^{3}$H]methylglucose as a model for small molecules with AT-II-induced hypertension from about 100 to about 150 mmHg. We also examined the effect of the artificial hypertension on the various side effects at or near lethal dose of SMANCS.

### Materials and methods

### *Animals and tumour*

We used female Wistar/Imamichi rats (200-250 g), bearing Walker 256 carcinoma. Walker 256 carcinoma cells were maintained by serial i.p. passage. Cells ($2 \times 10^6$) were injected i.p. or s.c. into the rats, and 5 days later the drug treatments were conducted. Usually, bloody ascites and a solid tumour on the omentum weighing about 1 g had developed 5 days later. This will therefore be a good model for peritoneal dissemination of tumour.

### *Drugs and chemicals*

AT-II (human) was purchased from Peptide Institute Inc., Osaka, Japan. $^{51}CrCl_3$ and [$^{3}$H]methylglucose were purchased from ICN Biochemicals Inc., Costa Mesa, CA, USA.

Correspondence: H. Maeda.
*Present address: Division of Cell Growth and Regulation, Dana-Farber Cancer Institute, Boston; Department of Biological Chemistry and Molecular Pharmacology, Harvard Medical School, Boston, MA, USA.
Received 19 November 1992; and in revised form 4 January 1993.

SMANCS, styrene-maleic acid copolymer-conjugated neocarzinostatin (Maeda *et al.*, 1979*a*,*b*; Takeshita *et al.*, 1982; Maeda *et al.*, 1984; Maeda *et al.*, 1985) was a generous gift from Kuraray Co., Ltd., Osaka, Japan. $^{51}$Cr-labelled bovine serum albumin ([$^{51}$Cr]BSA) was prepared by modifying the amino groups of bovine serum albumin (BSA) with anhydride of diethylenetriaminepentaacetic acid (DTPA) (protein/DTPA molar ratio = 1/20) to form a DTPA-BSA conjugate. DTPA was then chelated with $^{51}Cr^{3+}$ as described previously (Khaw *et al.*, 1980; Hnatowich *et al.*, 1982; Matsumura & Maeda, 1986). Antibody to the neocarzinostatin portion of SMANCS for quantification of SMANCS was kindly donated by Pola Chemical Co. Ltd., Tokyo, Japan. All other drugs and chemicals were from commercial sources.

### *Administration and quantification of drug*

Anesthetised rats by s.c. administration of pentobarbital sodium (30 mg kg$^{-1}$) were used as usual. [$^3$H]Methylglucose ($2 \times 10^6$ d.p.m./rat), [$^{51}$Cr]BSA ($1 \times 10^6$ d.p.m./rat), or SMANCS (10 mg kg$^{-1}$) was injected i.v. via the tail vein of rats under AT-II-induced hypertensive conditions (140–160 mmHg). The normotensive state (90–110 mmHg) was altered by infusion of AT-II via the tail vein at a rate of 2.0–4.0 μg kg$^{-1}$ min$^{-1}$, and the elevated blood pressure was maintained for 15 min during injection of the drugs. The blood pressure was monitored with an automatic tonometer (PS-200, Riken Kaihatsu Co., Ltd., Tokyo, Japan). When the infusion of AT-II stopped, the blood pressure returned to normal values in 5–10 min.

Rats were killed by i.v. administration of a lethal dose of pentobarbital sodium (150 mg kg$^{-1}$) at 15, 20, 60, and 360 min after drug administration to remove the tumour, the bone marrow, the small intestine, and blood to determine the drug concentration in these specimens. Concentrations of [$^3$H]methylglucose and [$^{51}$Cr]BSA were determined by counting the radioactivity as usual and that of SMANCS by enzyme-linked immunosorbent assay (ELISA) using anti-neocarzinostatin antibody and horseradish peroxidase-linked anti-mouse immunoglobulin antibody.

### *Evaluation of toxicities*

SMANCS at a dose giving 50% lethality ($LD_{50}$) (2 mg kg$^{-1}$) was injected i.v. via the tail vein of normal rats without tumour under hypertensive or normotensive conditions. The AT-II infusion was continued for 15 min as described. After 24 h, body weight was measured and blood was collected for determination of platelet and white blood cell (WBC) counts. The frequency and severity of diarrhoea were also recorded.

### Results

### *Distribution of [$^3$H]methylglucose*

Distribution of the radioactivity in the tumour and the bone marrow was quantified at 15, 20, or 60 min after i.v. administration of [$^3$H]methylglucose under hypertensive or normotensive conditions. As shown in Table I, the delivery of [$^3$H]methylglucose to tumour tissue at 15 min after administration was increased 20–25% by artificial hypertension. In particular, the amount of radioactivity in subcutaneous tumour was significantly increased by 1.26-fold, which was observed only at 15 min after injection. However, no advantage of artificial hypertension compared with normotensive conditions was observed when the values were evaluated at 60 min after injection. The radioactivity of [$^3$H]methylglucose in tumour tissue decreased with time regardless of the blood pressure at which the drug was administered.

AT-II-induced hypertension had little effect also on the distribution of [$^3$H]methylglucose in the bone marrow and blood at 20 or 60 min after injection.

### *Distribution of [$^{51}$Cr]BSA and SMANCS*

Results to reveal the effect of AT-II-induced hypertension on the distribution of the macromolecule [$^{51}$Cr]BSA to tumour and normal tissues are shown in Table II. The radioactivities in the omentum tumour, the ascites, the bone marrow, and of the blood at 1 and 6 h after i.v. administration of [$^{51}$Cr]BSA under normotensive and induced hypertensive conditions are given. Delivery of [$^{51}$Cr]BSA to tumour tissue and to the ascites increased by 1.8 and 1.3-fold, respectively, under hypertensive compared with normotensive conditions at 1 h. In contrast to the situation with [$^3$H]methylglucose, the amount of [$^{51}$Cr]BSA that was deposited in these tissues increased with time, and the drug concentration in the tumour increased significantly under hypertensive conditions compared with that of the normotensive groups. The higher values in the tumour tissue were retained even at 6 h after administration.

It should be noted that AT-II-induced hypertension resulted in less distrubtion of [$^{51}$Cr]BSA to the bone marrow and the small intestine (64–74% of that under normotensive conditions at 6 h). The values for the normal tissues showed

**Table I** Accumulation of [$^3$H]methylglucose in tumour and bone marrow after i.v. administration under AT-II-induced hypertensive conditions in rats

| *Specimen and time after injection* | *% Of injected dose/g tissue*[a] *Normotension* | *Hypertension* | *Ratio of distribution: hypertension/normotension (fold increased)* |
|---|---|---|---|
| Tumour, omentum | | | |
| 15 min | 1.04 ± 0.22 | 1.25 ± 0.17 | 1.20 |
| 60 min | 0.94 ± 0.13 | 1.04 ± 0.33 | 1.10 |
| Tumour, s.c. | | | |
| 15 min | 1.15 ± 0.15 | 1.45 ± 0.23[b] | 1.26[b] |
| 60 min | 0.85 ± 0.19[c] | 0.81 ± 0.16[c] | 0.96 |
| Bone marrow | | | |
| 20 min | 1.58 ± 0.48 | 1.54 ± 0.32 | 0.97 |
| 60 min | 1.45 ± 0.51 | 1.70 ± 0.17 | 1.11 |
| Blood | | | |
| 20 min | 1.08 ± 0.27 | 1.04 ± 0.26 | 0.96 |
| 60 min | 1.09 ± 0.37 | 1.12 ± 0.24 | 1.03 |

[a]Results are expressed as mean values ± s.d. [$^3$H]methylglucose ($2 \times 10^6$ d.p.m.) was injected i.v. during normotensive (90–110 mmHg) or AT-II induced hypertensive (140–160 mmHg) conditions. Infusion of AT-II was maintained to keep the hypertensive state for 15 min after injection of [$^3$H]methylglucose. Rats were killed at the indicated time after injection. Tumour, s.c. indicates tumour cells injected subcutaneously. Four animals were used for each group and each animal was inoculated with two s.c. and one omentum tumour. [b]Significantly different from the value obtained under normotensive conditions ($P < 0.01$, Student's *t*-test). [c]Significantly lower than the value obtained at 15 min ($P < 0.01$, Student's *t*-test).

**Table II** Accumulation of [$^{51}$Cr]BSA in tumour and other tissues after i.v. administration under AT-II-induced hypertensive conditions in rats

| *Specimen and time after injection* | *% Of injected dose/g tissue*[a] *Normotension* | *Hypertension* | *Ratio of distribution: hypertension/normotension (fold increased)* |
|---|---|---|---|
| Tumour, omentum | | | |
| 1 h | 0.26 ± 0.05 | 0.46 ± 0.11[b] | 1.77[b] |
| 6 h | 0.58 ± 0.09[c] | 0.70 ± 0.10[c] | 1.21 |
| Ascites | | | |
| 1 h | 0.48 ± 0.13 | 0.64 ± 0.04[b] | 1.33[b] |
| 6 h | 1.70 ± 0.44[c] | 2.07 ± 0.22[c] | 1.22 |
| Bone marrow | | | |
| 1 h | 0.23 ± 0.02 | 0.19 ± 0.02[b] | 0.83[b] |
| 6 h | 0.09 ± 0.02[c] | 0.06 ± 0.01[b,c] | 0.74[b] |
| Small intestine | | | |
| 1 h | 0.25 ± 0.01 | 0.23 ± 0.00 | 0.92 |
| 6 h | 0.31 ± 0.10 | 0.20 ± 0.06 | 0.64 |
| Blood | | | |
| 1 h | 4.64 ± 0.20 | 4.40 ± 0.43 | 0.95 |
| 6 h | 2.26 ± 0.53[c] | 2.05 ± 0.29[c] | 0.91 |

[a]Results are expressed as mean values ± s.d. [$^{51}$Cr]BSA (1 × $10^6$ d.p.m.) was injected i.v. during normotensive (90–110 mmHg) or AT-II-induced hypertensive (140–160 mmHg) conditions. Infusion of AT-II continued for 15 min after injection of [$^{51}$Cr]BSA. Rats were killed at 1 and 6 h after injection. Three or four rats were used for each group with tumour in the omentum. [b]Significantly higher or lower than the value under normotensive conditions ($P<0.05$, Student's *t*-test). [c]Significantly higher or lower than the value obtained at 1 h ($P<0.05$, Student's *t*-test).

no increase in the amount of the drug with time. Similar results were obtained when we examined the distribution of Evans blue, which binds to and behaves like albumin (Maeda *et al.*,1992): i.e. there was less accumulation in the bone marrow after i.v. injection under the same conditions described above by the EPR effect (data not shown).

We also tested the effect of AT-II-induced hypertension on the distribution of the macromolecular anticancer agents, SMANCS (16,000 Da) (Maeda *et al.*, 1979*a*; Maeda *et al.*, 1985), which is known to bind to albumin in the blood stream and hence behaves similar to albumin (Kobayashi *et al.*, 1988). Table III shows the concentration of SMANCS in the tumour, the bone marrow, and the small intestine at 1 and 6 h after injection, as determined by ELISA. The distribution of SMANCS to tumour was augmented significantly over time even under normotensive conditions, but it was enhanced even further under AT-II-induced hypertension. In contrast, distribution to bone marrow or small intestine tended to be reduced at 1 h after injection. Furthermore, enhanced intratumour concentration of the drug by using artificial hypertension was retained for 6 h, which is consistent with the results for [$^{51}$Cr]BSA. However, EPR effect alone facilitated much more (about 7-fold) accumulation of these macromolecules in tumour tissue over the bone marrow, or tumour over the intestine about 2-fold, where induced hypertension augmented EPR effect only about 2-fold.

The distribution ratios (tumour *vs* the normal tissues) of these macromolecules were calculated: namely, their concentration in tumour was divided by that in the bone marrow or the small intestine for each animal. As shown in Figure 1, selective accumulation of [$^{51}$Cr]BSA in the tumour was improved about 1.5–2-fold under AT-II-induced hypertension at both 1 and 6 h. Tumour-specific accumulation of SMANCS was also augmented by about 30–160% under artificial hypertension (Figure 2).

**Table III** Enhanced accumulation of SMANCS in tumour and reduction in normal tissues after i.v. administration under AT-II-induced hypertensive conditions in rats

| *Specimen and time after injection* | *% Of injected dose/g tissue*[a] *Normotension* | *Hypertension* | *Ratio of distribution: hypertension/normotension (fold increased)* |
|---|---|---|---|
| Tumour, s.c. | | | |
| 1 h | 0.29 ± 0.09 | 0.44 ± 0.17[b] | 1.63[b] |
| 6 h | 0.77 ± 0.51 | 0.95 ± 0.48[c] | 1.23 |
| Tumour, omentum | | | |
| 1 h | 0.26 ± 0.20 | 0.33 ± 0.28 | 1.27 |
| 6 h | 0.48 ± 0.28 | 0.88 ± 0.28[b,c] | 1.80[b] |
| Bone marrow | | | |
| 1 h | 0.24 ± 0.07 | 0.17 ± 0.10 | 0.71 |
| 6 h | 0.13 ± 0.06 | 0.14 ± 0.06 | 1.12 |
| Small intestine | | | |
| 1 h | 0.11 ± 0.05 | 0.07 ± 0.03 | 0.64 |
| 6 h | 0.05 ± 0.02 | 0.05 ± 0.01 | 0.98 |
| Blood | | | |
| 1 h | 3.03 ± 0.68 | 3.06 ± 0.73 | 1.01 |
| 6 h | 1.15 ± 1.05[c] | 1.27 ± 0.74[c] | 1.10 |

[a]Results are expressed as mean values ± s.d. SMANCS (10 mg $kg^{-1}$) was injected i.v. during normotensive (90–110 mmHg) or AT-II-induced hypertensive (140–160 mmHg) conditions. Infusion of AT-II continued for 15 min after injection of SMANCS. Five rats were used for each group and each rat had two s.c. and one omentum tumour. [b]Significantly different from the value under normotensive conditions ($P<0.05$, Student's *t*-test). [c]Significantly higher or lower than the value obtained at 1 h ($P<0.05$, Student's *t*-test).

**Figure 1** Tumour-specific increase in accumulation of [$^{51}$Cr]BSA after i.v. administration under AT-II-induced hypertensive conditions in rats. The concentration of [$^{51}$Cr]BSA in omentum tumour (Tu,om.) was divided by that in the bone marrow (B.marrow) or the small intestine (Sm.int.) for each animal to obtain the distribution ratio of [$^{51}$Cr]BSA; [$^{51}$Cr]BSA was administered under normotensive (□) or hypertensive (■) conditions. Each column shows the distribution ratio at 1 or 6 h after administration. Bars indicate s.d. Every value obtained at 6 h was significantly higher than that at 1 h in each condition ($P<0.05$, Student's $t$-test). # indicates that the value under hypertensive condition is significantly higher than that for the normotensive control ($P<0.05$, Student's $t$-test).

### *Toxic effects of a lethal dose of SMANCS under AT-II-induced hypertension*

We examined the effect of induced hypertension on the side effects of SMANCS at a lethal dose. As shown in Table IV, side effects such as leukopenia, diarrhoea, and body weight loss induced by high dose of SMANCS were significantly reduced under AT-II-induced hypertension. Thrombocytopenia was also reduced by this method, although the difference was statistically insignificant.

### Discussion

As reported by Suzuki *et al.* (1981) and Hori *et al.* (1991), AT-II-induced hypertension produces a selective increase in blood flow volume in tumour tissue because of the absence of autoregulation of tumour blood flow. In great contrast, normal tissue has an autoregulatory flow volume regardless of the blood pressure applied. An increase in blood flow is expected to result in increased drug delivery to tumour. On the basis of this principle, AT-II-induced hypertensive chemotherapy has been initiated in Japan (Sato *et al.*, 1981).

It is anticipated, however, that low-molecular-weight anticancer agents would traverse so freely out of the interstitial space back into the blood stream because of the concentration gradient that it may be difficult to retain higher drug concentrations in the tumour for an appreciable period after termination of AT-II infusion. By using high-molecular-weight drugs, we anticipated to circumvent this drawback knowing the EPR effect would take place for macromolecules. The results showed that artificial hypertension indeed induced the enhanced accumulation of [$^{3}$H]methylglucose in tumour tissue only at the early time point (15 min), however, this gain disappeared in 1 h, as shown in Table I. [$^{3}$H]Methylglucose may have been drained out of tumour tissue perhaps through both blood and lymphatic capillaries into the blood stream.

In contrast to these results with [$^{3}$H]methylglucose, a clear difference and advantage was obtained by the use of artificial hypertension in the intratumour concentrations of both macromolecules at 1 h, and which was well maintained even at 6 h after injection (Tables II and III). In general, macromolecules and lipids in the interstitial space are known to be recovered mainly by the lymphatics in normal tissues (Courtice, 1963), but they seemed unlikely to be cleared from tumour tissue effectively (Iwai *et al.*, 1984; Iwai *et al.*, 1987; Maeda *et al.*, 1984; Matsumura & Maeda, 1986). If the accumulated macromolecules in tumour could be drained by the operating lymphatics there would be no accumulation of the macromolecular drugs as in normal tissue. The results showed, however, that this appears to be unlikely because of the high accumulation in tumour (Tables II and III, Figures 1 and 2). This result suggests that little lymphatic drainage is taking place from tumour tissue. Furthermore, in contrast to

**Figure 2** Tumour-specific increase in accumulation of SMANCS after i.v. administration under AT-II-induced hypertensive conditions in rats. The concentration of SMANCS in subcutaneious tumour (Tu,s.c.) or omentum tumour (Tu,om.) was divided by that in the bone marrow (B.marrow) or the small intestine (Sm.int.) for each animal to obtain the distribution ratio of SMANCS. SMANCS was administered i.v. under normotensive (□) or hypertensive (■) conditions. Each column shows the distribution ratio at 1 or 6 h after administration. Bars indicate s.d. Every value obtained at 6 h was significantly higher than that at 1 h in each condition ($P<0.05$, Student's $t$-test). # indicates that the values under hypertensive conditions are significantly higher than those for the normotensive control ($P<0.05$, Student's $t$-test).

**Table IV** Reduced toxicity of SMANCS with AT-II-induced hypertension in healthy rats

| *Treatment* | *Increase in body wt (g)* | *Platelet ( × $10^4$ μl)* | *WBC ($mm^3$)* | *Diarrhoea (%)* |
|---|---|---|---|---|
| Control, normotensive | 1.5 ± 0.7 a | 98.9 ± 23.8 | 10733 ± 902 b b | 0 c |
| SMANCS, normotensive | − 7.4 ± 4.3 a a | 53.9 ± 8.8 | 3133 ± 306 b b | 80 c c |
| SMANCS + AT-II, hypertensive | 0.6 ± 0.9 a | 73.8 ± 26.3 | 4267 ± 231 b b | 0 c |

SMANCS (2 mg kg$^{-1}$) was injected i.v. under AT-II-induced hypertensive (140–160 mmHg) or normotensive (90–110 mmHg) conditions as described in Tables I–III. Values are means ± s.d. at 24 h after treatment. Five rats for each group.
a, b, and c Significantly different between the values indicated. $^aP<0.02$; $^bP<0.001$; $^cP<0.01$.

the intratumour concentration of [$^3$H]methylglucose, which decreased more rapidly with time (Table I), in intratumour concentration of [$^{51}$Cr]BSA and SMANCS, compared with that in normal tissue, increased with time (Figures 1 and 2) as previously observed (Maeda *et al.*, 1984; Matsumura & Maeda, 1986) and attributed to the *EPR effect.*

The results became more pronounced when the blood pressure was increased while drugs were administered (Tables II and III). Unlike small molecules, macromolecules and lipids themselves are tumouritropic because of the EPR effect, as reported previously (Maeda *et al.*, 1984; Matsumura & Maeda, 1986; Maeda & Matsumura, 1989; Maeda, 1991; Maeda *et al.*, 1992). Using another macromolecule (hydroxypropylmethacrylamide copolymer) developed by a joint effort of groups in the UK and Czechoslovakia, we also observed a time-dependent increase in intratumour concentration (EPR effect) (Seymour, L., Miyamoto, Y., *et al.*, unpublished).

We have also found another phenomenon that little lymphatic clearance of lipids (Konno *et al.*, 1984*a,b*; Iwai *et al.*, 1984; Maeda *et al.*, 1984; Iwai *et al.*, 1987) and macromolecules (Maeda *et al.*, 1984; Matsumura & Maeda, 1986; Maeda & Matsumura, 1989; Maeda, 1991) from solid tumours takes place.

The EPR effect can be attributed to a hypervascularised state resulting from tumour angiogenesis (Folkman & Klagsbrun, 1987), and incomplete vascular architecture and leakiness (Suzuki *et al.*, 1987; Skinner *et al.*, 1990). Furthermore, we found a mechanism that allows a high local concentration of bradykinin which enhances vascular permeability; in which tumour cells activate the Hageman factor/kallikrein/kinin cascade (Maeda *et al.*, 1988; Matsumura *et al.*, 1988; Matsumura *et al.*, 1990; Matsumura *et al.*, 1991). As a consequence, vascular permeability will be elevated. Another permeability-enhancing factor that would function in an additive manner to the kinin system is also known (Senger *et al.*, 1983; Dvorak *et al.*, 1985).

It is suggested that the major obstacle to the delivery of macromolecules to solid tumours is the elevated interstitial fluid pressure in tumour tissues (Jain, 1990). Our work described here indicates that AT-II-induced hypertension is an effective way for overcoming such an obstacle of intratumoural fluid pressure by increasing blood pressure/flow/vascular surface area in tumour tissue (Suzuki *et al.*, 1981; Hori *et al.*, 1983; Hori *et al.*, 1984), which may all enhance the convective influx of macromolecules into the tumour compartment out of the blood capillary. Present and previous observations of EPR effect or higher retention in tumour indicate reverse transfer of macromolecules and lipids does not seem to occur as a vascular barrier or characteristics.

The results described here also imply that this method may improve the delivery of monoclonal antibodies to tumour tissue. Several approaches have been examined to increase the tumour uptake of monoclonal antibodies so far (e.g. Smyth *et al.*, 1987; Kalofonos *et al.*, 1990; Cope *et al.*, 1990; Russel *et al.*, 1990). Recently we reported that ATI-II-induced hypertension combined with the use of kininase inhibitor enhanced the tumour localisation of a monoclonal antibody up to 200% of control (Noguchi *et al.*, 1992). Kininase inhibitor might enhance the vascular permeability by prolonging the action of kinin (Maeda *et al.*, 1988; Matsumura *et al.*, 1988; Matsumura *et al.*, 1990; Matsumura *et al.*, 1991).

The second advantage clarified in this study was the reduction in accumulation of the drug by normal tissues, such as the bone marrow and the small intestine, by using macromolecular drugs rather than small molecules in induced hypertension chemotherapy. AT-II-induced hypertension decreased the blood flow volume in the kidneys because of vascular constriction, resulting in reduced renal toxicity of cisplatin (Kuroiwa *et al.*, 1987). In general, however, arterial blood flow remains constant in normal tissues, in the range between 50 and 150 mmHg, by autoregulatory contraction of arterioles as described above (Suzuki *et al.*, 1981), with some exceptions such as the kidney (Kuroiwa *et al.*, 1987). It is consistent then that accumulation of [$^3$H]methylglucose in bone marrow was little affected by manipulating blood pressure, as shown in Table I. In contrast to the accumulation of [$^3$H]methylglucose, the accumulation of macromolecules represented by [$^{51}$Cr]BSA and SMANCS in the bone marrow was reduced under hypertensive conditions as shown in Tables II and III. A decrease in the distribution of macromolecules to normal tissues under AT-II-induced hypertension was confirmed by the reduction of the toxicity of SMANCS administered at a lethal dose (Table IV). Significant protection against leukopenia, diarrhoea, and loss of body weight was noted, reflecting the reduced delivery of SMANCS to these organs (Table III). Accordingly, AT-II-induced hypertension can improve the therapeutic index of macromolecular anticancer agents.

This work has been supported by a Grant-in-Aid for Scientific Research from the Ministry of Education, Science and Culture (Monbusho) of Japan to H. Maeda for 1989–1992. We thank gratefully Dr A. Pardee of Dana Farber Cancer Institute for reading and discussing this manuscript.

### References

COPE, D.A., DEWHIRST, M.W., FRIEDMAN, H.S., BIGNER, D.D. & ZALUTSKY, M.R. (1990). Enhanced delivery of a monoclonal antibody F(ab')2 fragment to subcutaneous human glioma xenografts using local hyperthermia. *Cancer Res.*, **50**, 1803–1809.

COURTICE, F.C. (1963). The orgin of lipoprotein in lymph. In *Lymph and Lymphatic System*, Meyersen, H.S. (Chairman) pp. 89–126. Springfield, Charles C. Thomas: Illinois.

DVORAK, H.F., SENGER, D.R., DVORAK, A.M., HARVER, V.S. & MCDONAGH, J. (1985). Regulation of extravascular coagulation by microvascular permeability. *Science*, **227**, 1059–1061.

FOLKMAN, J. & KLAGSBRUN, M. (1987). Angiogenic factors. *Science*, **235**, 442–447.

HNATOWICH, D.J., LAYNE, W.W. & CHILDS, R.L. (1982). The preparation and labeling of DTPA-coupled albumin. *Int. J. Appl. Radiat. Isot.*, **33**, 327–332.

HORI, K., SUZUKI, M., ABE, I., SAITO, S. & SATO, H. (1983). Microocclusion technique for measurement of the microvascular pressure in tumor and subcutis. *Jpn. J. Cancer Res.*, **74**, 122–127.

HORI, K., SUZUKI, M., ABE, I., SAITO, S. & SATO, H. (1984). Increase in tumor vascular area due to increased blood flow by angiotensin II in rats. *J. Natl Cancer Inst.*, **74**, 453–459.

HORI, K., SUZUKI, M., TANDA, S., SAITO, S., SHINOZAKI, S. & ZHANG, Q.-H. (1991). Fluctuation in tumor blood flow under normotension and the effect of angiotensin II-induced hypertension. *Jpn. J. Cancer Res.*, **82**, 1309–1316.

IWAI, K., MAEDA, H. & KONNO, T. (1984). Use of oily contrast medium for selective drug targeting to tumor: enhanced therapeutic effect and X-ray image. *Cancer Res.*, **44**, 2115–2121.

IWAI, K., MAEDA, H., KONNO, T., MATSUMURA, Y., YAMASHITA, R., YAMASAKI, K., HIRAYAMA, S. & MIYAUCHI, Y. (1987). Tumor targeting by arterial administration of lipids: rabbit model with VX2 carcinoma in the liver. *Anticancer Res.*, **7**, 321–328.

JAIN, R.K. (1990). Physiological barriers to delivery of monoclonal antibodies and other macromolecules in tumors. *Cancer Res.* (Suppl.), **50**, 814s–819s.

KALOFONOS, H., ROWLINSON, G. & EPENETOS, A.A. (1990). Enhancement of monoclonal antibody uptake in human colon tumor xenografts following irradiation. *Cancer Res.*, **50**, 159–163.

KHAW, B.A., FALLON, J.T., STRAUSS, H.W. & HARBER, F. (1980). Myocardial infarct imaging of antibodies to canine cardiac myosin with indium-111-diethylenetriamine pentaacetic acid. *Science*, **209**, 295–297.

KOBAYASHI, A., ODA, T. & MAEDA, H. (1988). Protein binding of macromolecular anticancer agent SMANCS: characterization of poly(styrene-co-maleic acid) derivatives as an albumin binding ligand. *J. Bioactive Compatible Polym.*, **3**, 319–333.

KONNO, T., MAEDA, H., IWAI, K., TASHIRO, S., MAKI, S., MOCHINAGA, M., HIRAOKA, T. & YOKOYAMA, I. (1984*a*). Effect of arterial administration of high molecular weight anticancer agent SMANCS with lipid lymphographic agent on hepatoma: a preliminary report. *Eur. J. Cancer Clin. Oncol.*, **19**, 1053–1065.

KONNO, T., MAEDA, H., IWAI, K., MAKI, S., TASHIRO, S., UCHIDA, M. & MIYAUCHI, Y. (1984*b*). Selective targeting of anticancer drug and simultaneous image enhancement in solid tumors by arterially administered lipid contrast medium. *Cancer*, **54**, 2367–2374.

KUROIWA, T., AOKI, K., TANIGUCHI, S., HASUDA, K. & BABA, T. (1987). Efficacy of two-route chemotherapy using cis-diamminedichloroplatinum(II) and its antidote, sodium thiosulfate, in combination with angiotensin II in a rat limb tumor. *Cancer Res.*, **47**, 3618–3623.

MAEDA, H., TAKESHITA, J. & KANAMARU, R. (1979*a*). A lipophilic derivative of neocarzinostatin. A polymer conjugation of an antitumor protein antibiotic. *Int. J. Pept. Protein Res.*, **14**, 81–87.

MAEDA, H., TAKESHITA, J., KANAMARU, R., SATO, H., KHATOH, J. & SATO, H. (1979*b*). Antimetastatic and antitumor activity of a derivative of neocarzinostatin: an organic solvent- and water-soluble polymer-conjugated protein. *Gann (Jpn. J. Cancer Res.)*, **70**, 601–606.

MAEDA, H., MATSUMOTO, T., KONNO, T., IWAI, K. & UEDA, M. (1984). Tailor-making of protein drugs by polymer conjugation for tumor targeting: a brief review on smancs. *J. Protein Chem.*, **3**, 181–193.

MAEDA, H., UEDA, M., MORINAGA, T. & MATSUMOTO, T. (1985). Conjugation of poly(styrene-co-maleic acid) derivatives to the antitumor protein neocarzinostatin: pronounced improvements in pharmacological properties. *J. Med. Chem.*, **28**, 455–461.

MAEDA, H., MATSUMURA, Y. & KATO, H. (1988). Purification and identification of hydroxyprolyl$^3$-bradykinin in ascitic fluid from a patients with gastric cancer. *J. Biol. Chem.*, **263**, 16051–16054.

MAEDA, H. & MATSUMURA, Y. (1989). Tumoritropic and lymphotropic principles of macromolecular drugs. *Crit. Rev. Ther. Drug Carrier Syst.*, **6**, 193–210.

MAEDA, H. (1991). SMANCS and polymer-conjugated macromolecular drugs: advantages in cancer chemotherapy. *Adv. Drug Delivery Rev.*, **6**, 181–202.

MAEDA, H., SEYMOUR, L.W. & MIYAMOTO, Y. (1992). Conjugation of anticancer agents and polymers: advantages of macromolecular therapeutics *in vivo*. *Bioconjugate Chem.*, **3**, 351–362.

MATSUMURA, Y. & MAEDA, H. (1986). A new concept for macromolecular therapeutics in cancer chemotherapy: mechanisms of tumoritropic accumulation of proteins and the antitumor agent smancs. *Cancer Res.*, **46**, 6387–6392.

MATSUMURA, Y., KIMURA, M., YAMAMOTO, T. & MAEDA, H. (1988). Involvement of the kinin-generating cascade in enhanced vascular permeability in tumor tissue. *Jpn. J. Cancer Res.*, **79**, 1327–1334.

MATSUMURA, Y., KATO, H. & MAEDA, H. (1990). Degradation pathway of kinins in tumor ascites and inhibition by kininase inhibitors: analysis by HPLC. *Agents Actions*, **29**, 172–180.

MATSUMURA, Y., MARUO, K., KIMURA, M., YAMAMOTO, T., KONNO, T. & MAEDA, H. (1991). Kinin-generating cascade in advanced cancer patients and *in vitro* study. *Jpn. J. Cancer Res.*, **82**, 732–741.

NOGUCHI, A., TAKAHASHI, T., YAMAGUCHI, T., KITAMURA, K., NOGUCHI, A., TSURUMI, H., TAKASHINA, K. & MAEDA, H. (1992). Enhanced tumor localization of monoclonal antibody by treatment with kininase II inhibitor and angiotensin II. *Jpn. J. Cancer Res.*, **83**, 240–243.

RUSSEL, S.M., KRAUER, K.G., MCKENZIE, F.C. & PIETERSZ, G.A. (1990). Effect of tumor necrosis factor on the antitumor efficacy and toxicity of aminopterine-monoclonal antibody conjugates: parameters for optimization of therapy. *Cancer Res.*, **50**, 6028–6033.

SATO, H., SATO, K., SATO, Y., ASAMURA, M., KANAMARU, R., SUGIYAMA, Z., KITAHARA, T., WAKUI, A., SUZUKI, M., HORI, K., ABE, I., SAITO, S., & SATO, H. (1981). Induced hypertension chemotherapy of cancer patients by selective enhancement of drug delivery to tumor tissue with angiotensin II. *Sci. Rep. Inst. Tohoku Univ.* **Ser. C 28**, 32–44.

SENGER, D.R., GALLI, S.J. & DVORAK, A.K. (1983). Tumor cells secrete a vascular permeability factor that promotes accumulation of ascitic fluid. *Science*, **219**, 983–985.

SKINNER, S.A., TUTTON, P.J.M. & O'BRIEN, P.E. (1990). Microvascular architecture of experimental colon tumors in the rat. *Cancer Res.*, **50**, 2411–2417.

SMYTH, M.J., PIETERSZ, G.A. & MCKENZIE, F.C. (1987). Use of vasoactive agents to increase tumor perfusion and the antitumor efficacy of drug-monoclonal antibody conjugates. *J. Natl Cancer Inst.*, **79**, 1367–1373.

SUZUKI, M., HORI, K., ABE, I., SAITO, S. & SATO, H. (1981). A new approach to cancer chemotherapy: a selective enhancement of tumor blood flow with angiotensin II. *J. Natl Cancer Inst.*, **67**, 663–669.

SUZUKI, M., TAKAHASHI, T. & SATO, T. (1987). Medial regression, and its functional significance in tumor-supplying host arteries. *Cancer*, **59**, 444–450.

TAKESHITA, J., MAEDA, M. & KANAMARU, R. (1982). *In vitro* mode of action, pharmacokinetics, and organ specificity of poly (maleic acid-styrene)-conjugated neocarzinostatin, SMANCS. *Gann (Jpn. J. Cancer Res.)*, **73**, 278–284.

WAKUI, A. & SATO, H. (1984). Clinical studies on induced hypertension chemotherapy based on functional characteristics of microcirculation of tumor vessels. *Jpn. J. Cancer Chemother.*, **11**, 741–749 (in Japanese).